# Access to care, treatment pathways, and outcomes of endovascular treatment for aneurysmal subarachnoid hemorrhage in Kazakhstan: a retrospective cohort study

**DOI:** 10.3389/fradi.2026.1845453

**Published:** 2026-06-26

**Authors:** Mynzhylky Berdikhojayev, Aiman Maidan, Karlygash Toguzbaeva, Dimash Davletov, Abzal Zhumabekov, Shayakhmet Makhanbetkhan, Daulet Suieumbetov, Rauan Aiteli, Maxat Mussabekov, Roger Barranco Pons, Marat Sarshayev

**Affiliations:** 1 National Hospital of the Medical Center of the Presidential Affairs Administration of the Republic of Kazakhstan, Almaty, Kazakhstan; 2 Department of Interventional Neuroradiology and Neurosurgery, Instituto Médico ENERI, Clínica La Sagrada Familia, Buenos Aires, Argentina; 3 Asfendiyarov Kazakh National Medical University, Almaty, Kazakhstan; 4 Al Qassimi Hospital, Sharjah, United Arab Emirates

**Keywords:** access to care, aneurysmal subarachnoid hemorrhage, global neurosurgery, health system determinants, upper-middle-income countries

## Abstract

**Background:**

Aneurysmal subarachnoid hemorrhage (aSAH) remains a major cause of mortality and disability worldwide. Outcomes after aSAH are influenced not only by hemorrhage severity and aneurysm characteristics but also by patient comorbidities, healthcare organization, and access to specialized neurovascular care. This study evaluated clinical and health-system determinants of functional outcome after aSAH in Kazakhstan.

**Methods:**

We conducted a single-center retrospective cohort study of 483 patients with ruptured intracranial aneurysms treated endovascularly at a national tertiary neurovascular center (2016–2024). Early aneurysm occlusion was defined as treatment within 24 h of symptom onset. For cohort analyses, patients treated within 14 days were classified as the acute group, whereas those treated after 14 days comprised the delayed-treatment group, including both referral-related delays and deferred treatment after clinical stabilization. Demographic, clinical, radiological, and procedural variables were analyzed. Functional outcomes were assessed using the modified Rankin Scale (mRS) at discharge, 6 months, and 12 months. Favorable recovery was defined as mRS ≤2 at 6–12 months.

**Results:**

The mean age was 52.6 years, and 65.4% were female. Rural residence was associated with delayed access to treatment (60.9% vs. 42.9%, *p* < 0.001). In the representative 2021 referral cohort, the median interval from SAH onset to arrival at the tertiary neurovascular center was 26 days (IQR 9–54), reflecting substantial referral and transfer delays within the centralized healthcare system. In multivariable analysis, periprocedural complications (adjusted OR 8.68, *p* < 0.001) and ischemic heart disease (adjusted OR 3.07, *p* = 0.012) were independently associated with mRS 3–6, whereas higher admission Glasgow Coma Scale scores were protective (adjusted OR 0.64, *p* < 0.001). Aneurysm morphology and traditional SAH severity scales were not independently associated with long-term outcome.

**Conclusions:**

Functional outcome after aneurysmal SAH within a centralized upper-middle-income neurovascular system was driven primarily by neurological status, systemic comorbidity, and procedural safety rather than aneurysm anatomy. Given the treatment-based referral design and survivorship bias inherent in tertiary transfer cohorts, these findings suggest that centralized systems facilitate access to specialized neurovascular care for patients reaching tertiary centers. Strengthening complication prevention, cardiovascular risk reduction, and referral pathways may improve neurovascular outcomes.

## 1 Introduction

Spontaneous aneurysmal subarachnoid hemorrhage (aSAH) remains a major cause of mortality and long-term neurological disability worldwide and is most commonly caused by rupture of intracranial aneurysms (1). Despite advances in microsurgical and endovascular techniques, as well as improvements in neurocritical care, outcomes after aSAH remain highly heterogeneous, with substantial residual morbidity even among successfully treated patients. The burden of aSAH is particularly relevant in low- and middle-income healthcare systems, where disparities in emergency transport, referral infrastructure, neurovascular workforce distribution, and access to referral-center treatment may significantly influence survival and recovery (2). Geographic inequities in access to tertiary neurovascular care remain an important global neurosurgical challenge, especially in countries with centralized healthcare structures and large territorial catchment areas (3).

Most contemporary evidence regarding predictors of outcome after aSAH originates from high-income countries with mature neurovascular networks and relatively uniform access to acute aneurysm treatment (4, 5). In contrast, data from upper-middle-income countries remain comparatively limited, particularly regarding the interaction between patient comorbidities, treatment timing, referral pathways, and health-system organization (6). In centralized healthcare systems, patients from rural or geographically remote regions frequently undergo initial stabilization at local hospitals before delayed transfer to definitive aneurysm treatment. Such referral patterns may substantially influence treatment strategy, perioperative risk, and observed functional outcomes independently of aneurysm anatomy or hemorrhage severity (7).

Kazakhstan represents a typical upper-middle-income neurovascular model characterized by advanced expertise concentrated within a limited number of tertiary referral centers serving large geographic regions (8). During the study period, endovascular resources, angiography availability, detachable coils, and neurointerventional specialists were not uniformly available across all regional hospitals, necessitating transfer of many patients to centralized neurovascular centers for definitive treatment. This structure creates substantial variability in treatment timing and referral pathways while also providing a unique opportunity to evaluate the interaction between geographic access, centralized care, and functional recovery after aSAH (9).

The present study therefore aimed to: (1) describe disparities in referral patterns and treatment timing between urban and rural populations with aneurysmal SAH; (2) identify demographic, clinical, procedural, and health-system predictors of functional outcome following endovascular treatment; and (3) evaluate whether a centralized tertiary neurovascular referral system may facilitate access to specialized care across geographically diverse populations within an upper-middle-income healthcare setting.

## 2 Methods

### 2.1 Study design and setting

A retrospective observational study was conducted including all patients with aneurysmal subarachnoid hemorrhage (aSAH) treated at JSC National Hospital of the Medical Center of the Presidential Affairs Administration of the Republic of Kazakhstan between January 2016 and December 2024.

Inclusion criteria were: (1) confirmed aneurysmal subarachnoid hemorrhage, (2) angiographically verified ruptured intracranial aneurysm, and (3) endovascular treatment performed at the tertiary center. Exclusion criteria included: (1) non-aneurysmal SAH, (2) incomplete clinical or imaging data, (3) absence of endovascular treatment, and (4) patients treated exclusively at outside institutions without transfer. Cases treated exclusively with microsurgical clipping were not included, reflecting the center's endovascular treatment profile.

### 2.2 Residential classification

Residential status was classified according to national administrative definitions. Urban residence was defined as living in Almaty or in officially designated urban settlements (cities and towns), whereas rural residence included villages, districts, and non-metropolitan settlements outside major urban centers.

### 2.3 Treatment timing, Pre-treatment management, and transfer logistics

In Kazakhstan, access to acute endovascular aneurysm treatment remains heterogeneous across regions. During the study period, several regional hospitals lacked continuous availability of neurointerventional physicians, angiography suites, or endovascular materials such as detachable coils, necessitating transfer to tertiary neurovascular centers for definitive treatment. In accordance with AHA/ASA recommendations, “early aneurysm occlusion” was defined as treatment performed within 24 h after symptom onset. For cohort stratification, patients treated within 14 days after rupture were classified as the acute treatment group, whereas patients treated more than 14 days after hemorrhage were classified as the delayed treatment group. The delayed group included both patients experiencing referral-related logistical delays and patients undergoing delayed treatment after stabilization to facilitate reconstructive or stent-assisted strategies.

Treatment timing was analyzed as a system-level variable reflecting both clinical decision-making and referral logistics.

Rebleeding prior to aneurysm occlusion was systematically assessed. In-hospital rebleeding was defined as acute neurological deterioration accompanied by radiological confirmation of recurrent hemorrhage before definitive treatment. These events were recorded and considered in the interpretation of treatment timing and clinical outcomes.

Before definitive intervention, patients received standardized stabilization according to institutional neurocritical care protocols. This included neurological monitoring, blood pressure control, routine administration of nimodipine, and supportive care measures. Additional interventions, such as airway protection or cerebrospinal fluid diversion, were performed when clinically indicated. Emergency vascular imaging was obtained to confirm aneurysm characteristics and guide treatment strategy.

Patients from remote regions were typically initially managed at regional hospitals, where primary stabilization was performed prior to transfer. Transportation to the tertiary center was carried out via ground or air medical services depending on geographic distance and patient condition. During transfer, patients received continuous supportive care in accordance with emergency medical protocols. Importantly, in many referring regions, access to specialized neurosurgical or neurointerventional care was limited or unavailable. As a result, patients were transferred not only due to geographic distance but also due to the absence of local capacity for definitive aneurysm treatment. Consequently, the referral process reflects structural disparities in healthcare access rather than purely logistical delay.

Given the centralized referral structure, the study cohort represents a treatment-based population and is subject to inherent selection bias. Patients transferred from remote areas likely represent a subset of individuals who survived the initial hemorrhagic event and were considered suitable for transfer. This reinforces that the cohort reflects patients who successfully reached a tertiary center with treatment capability, while individuals without access to referral pathways or those who deteriorated prior to transfer are not captured. To account for this, residential status and treatment timing were incorporated into multivariable analyses; however, the findings should be interpreted as determinants of outcome among patients who accessed definitive aneurysm treatment rather than population-level SAH outcomes.

### 2.4 Referral pathways and geographic representation

To illustrate the national referral structure and geographic catchment of the study center, the referral system was mapped using data from the year 2021 as a representative example. This year was selected due to the availability of complete referral documentation and stable organizational structure of emergency neurovascular care. The mapping demonstrates the geographic distribution of patients referred to the tertiary center from urban and rural regions and reflects routine referral patterns rather than temporal trends. The referral pathway visualization was intended solely to contextualize healthcare access and was not incorporated into regression modeling or hypothesis testing. The delayed treatment cohort represented a heterogeneous population combining patients transferred after logistical or geographic delays with patients in whom delayed intervention was intentionally selected following stabilization to facilitate reconstructive or stent-assisted treatment strategies. Accordingly, observed associations between treatment timing and functional outcome should be interpreted cautiously.

### 2.5 Data collection

Demographic, clinical, radiological, procedural, and follow-up data were extracted from institutional electronic medical records and imaging archives. Variables were selected to capture not only aneurysm- and procedure-related characteristics but also systemic, behavioral, and social factors relevant to population-level outcomes. The study was reported in accordance with STROBE recommendations for observational cohort studies.

### 2.6 Clinical and radiological variables

Patient-level variables included age, sex, smoking status, arterial hypertension, ischemic heart disease (IHD), diabetes mellitus (DM), and acute kidney injury (AKI).

Aneurysm characteristics included arterial location, laterality, morphology, maximal diameter, and neck configuration. Aneurysm size was categorized as small (<7 mm), medium (7–15 mm), large (15–25 mm), or giant (>25 mm). Broad-neck aneurysms were defined as having a neck diameter ≥4 mm or a dome-to-neck ratio <2, consistent with established criteria (5).

Aneurysm morphology was classified as saccular, blister, fusiform, dissecting, or fenestrated based on angiographic features (5, 10, 11). Daughter sacs (blebs) were recorded as secondary dome irregularities or lobulations identified on three-dimensional angiography (11).

### 2.7 Clinical severity and complications

Baseline neurological severity was assessed using the Hunt–Hess scale, World Federation of Neurosurgical Societies (WFNS) grade, Fisher grade, and admission Glasgow Coma Scale (GCS). These variables were included in multivariable models to adjust for initial hemorrhage severity and neurological injury. Delayed (treatment after 14 days) treatment does not equal delay due to system failure, but often reflects intentional stabilization for safer reconstructive therapy. Periprocedural and in-hospital complications were systematically recorded, including delayed cerebral ischemia (DCI), vasospasm, ischemic and hemorrhagic stroke, infections, and cardiopulmonary events. Vasospasm was defined as radiographic arterial narrowing, whereas DCI was defined as clinical or imaging-confirmed cerebral ischemia attributable to impaired cerebral perfusion. Ischemic stroke unrelated to vasospasm or DCI was analyzed separately (12). Outcome-modifying complications were defined as new neurological or systemic events occurring during hospitalization that altered the patient's baseline clinical course.

### 2.8 Antiplatelet management

For stent reconstructive endovascular procedures, for recanalized aneurysms previously treated at other centers, antiplatelet therapy was administered according to institutional protocol and individualized based on clinical urgency. Dual antiplatelet therapy with aspirin and a P2Y12 inhibitor (clopidogrel or prasugrel) was initiated when stent placement was planned. In cases requiring unanticipated or rescue stent deployment, antiplatelet therapy was initiated peri-procedurally or immediately post-procedure. Subsequent maintenance therapy and adjustments were guided by clinical status and treating physician discretion.

### 2.9 Follow-up, recanalization, and re-treatment

Follow-up data were obtained through outpatient visits, imaging review, and structured telephone assessment when in-person evaluation was unavailable. All patients underwent standardized follow-up imaging with magnetic resonance imaging and angiography (MRI/MRA) at approximately 3 months after treatment, followed by digital subtraction angiography (DSA) at 6 months. Functional outcomes were evaluated using the modified Rankin Scale (mRS) at discharge, 6 months, and 12 months. The primary endpoint was 12-month functional outcome assessed by mRS. Six-month follow-up data were used only when 12-month follow-up was unavailable.

Recanalization was defined as angiographic reopening or progression of residual aneurysm filling during follow-up. Patients with recanalization without rebleeding were considered for endovascular re-treatment, which was performed based on anatomical findings and clinical judgment.

### 2.10 Socioeconomic and environmental variables

Residential status was classified as urban or rural according to national administrative definitions. Occupational stress was assessed using validated instruments such as the Perceived Stress Scale (PSS-10) when available (13). In cases where standardized assessment was unavailable, occupational responsibility was retrospectively categorized as low or high stress based on documented job demands, acknowledging this as a limitation due to incomplete historical data. Information on education level, income, and healthcare access was not consistently available and was therefore not included in the analysis. Given the retrospective nature of socioeconomic data, occupational stress classification was used as an exploratory variable and interpreted cautiously in multivariable analyses.

### 2.11 Statistical analysis

Continuous variables were expressed as mean ± standard deviation, and categorical variables as frequencies and percentages. Candidate variables were selected based on clinical relevance and univariate association (*p* < 0.10). To reduce overfitting, the final model was constructed using a stepwise backward selection approach while ensuring adequate events-per-variable ratio. Collinearity between severity scales was assessed using variance inflation factors, and only one severity metric was retained when multicollinearity was detected. Group comparisons were performed using the Chi-square test or Fisher's exact test when appropriate. For cells with small expected counts, Monte Carlo simulation was applied to ensure reliable estimation. Variables demonstrating *p* < 0.10 in univariable analyses were entered into multivariable logistic regression models using backward stepwise selection. Multicollinearity was assessed using variance inflation factor (VIF) analysis prior to final model construction. Odds ratios (ORs) and 95% confidence intervals (CIs) were reported for significant associations. Missing data were infrequent (<2%) and complete-case analysis was performed without imputation. All *p*-values were reported to three decimal places, except for values <0.001, which were reported as *p* < 0.001. All statistical analyses were performed using R software for statistics (version 4.5.1).

### 2.12 Ethical approval

All procedures performed in studies involving human participants were in accordance with the ethical standards of the Institutional Research Ethics Committee of the JSC National Hospital of the Medical Center of the Presidential Affairs Administration of the Republic of Kazakhstan and with the 1964 Helsinki Declaration and its later amendments. This retrospective study was approved under protocol number 38, dated 17/02/2025.

### 2.13 Consent to participate

Waiver of informed consent was approved by ethics committee due to retrospective design.

## 3 Results

### 3.1 Study population and care pathways

A total of 483 patients with ruptured intracranial aneurysms were included (2016–2024). The cohort was predominantly female (65.4%), with a mean age of 52.6 years (range 20–84). Early aneurysm occlusion within 24 h after symptom onset was achieved in 78 patients (16.1%). Acute treatment performed between 24 h and 14 days after rupture was observed in 106 patients (21.9%), whereas 299 patients (61.9%) underwent delayed treatment more than 14 days after rupture. Baseline patient characteristics are summarized in Table 1.

**Table 1:** Baseline characteristics.

Demographics	*n* (%) or mean ± SD
Age, years	52.6 ± 11.6 (20–84)
Female	316 (65.4)
Male	167 (34.6)
Urban residence	276 (57.1)
Rural residence	207 (42.9)
Smoking	78 (16.1)
Arterial hypertension	443 (91.7)
Diabetes mellitus	37 (7.7)
Ischemic heart disease/CHF	81 (16.8)
Clinical severity	
Hunt–Hess I	367 (76.0)
Hunt–Hess II	29 (6.0)
Hunt–Hess III	43 (8.9)
Hunt–Hess IV	44 (9.1)
WFNS I	365 (75.6)
WFNS II	25 (5.2)
WFNS III	41 (8.5)
WFNS IV	52 (10.8)
Fisher grade 1	334 (69.2)
Fisher grade 2	7 (1.5)
Fisher grade 3	17 (3.5)
Fisher grade 4	125 (25.9)
Admission GCS, median (IQR)	14 (13–15)
Aneurysm characteristics	
Anterior circulation	460 (95.2)
Posterior circulation	23 (4.8)
Right-sided	141 (29.2)
Left-sided	143 (29.6)
Midline (ACom/BA/ACA complex)	138 (28.6)
Multiple aneurysms	61 (12.6)
Small (<7 mm)	347 (71.6)
Medium (7–15 mm)	96 (19.9)
Large (15–25 mm)	26 (5.6)
Giant (>25 mm)	14 (2.9)
Saccular	461 (95.5)
Non-saccular (fusiform/blister/dissecting/other)	22 (4.5)
Broad-neck aneurysm	161 (33.3%)
Treatment characteristics	
Acute treatment	184 (38.1)
Primary coiling alone	118 (24.4)
Balloon-assisted coiling	268 (55.5)
Stent-assisted coiling/reconstructive coiling	55 (11.4)
Flow diversion/stent monotherapy/parent vessel occlusion/WEB	42 (8.7)

Residence significantly influenced treatment timing. Urban patients were more likely to receive acute care (57.07%), whereas rural patients were disproportionately managed in delayed treatment settings (60.87%; *p* < 0.001), indicating geographic disparities in access to urgent neurovascular services (Table 2). Patients treated electively were slightly older (median 55 vs. 52 years; *p* = 0.016).

**Table 2:** Association of systemic comorbidities with functional outcome (mRS) and timing.

mRS at discharge					
Smoking	mRS 0–2	mRS 3–6	Total	*p*-value	Statistical test
Absent	357 (85.20%)	48 (75.00%)	405	0.039	Chi-square
Present	62 (14.80%)	16 (25.00%)	78		
Arterial hypertension (AH)				0.183	Chi-square
Absent	34 (8.11%)	6 (9.38%)	40		
AH Stage 1	30 (7.16%)	9 (14.06%)	39		
AH Stage 2	48 (11.46%)	4 (6.25%)	52		
AH Stage 3	307 (73.27%)	45 (70.31%)	352		
Diabetes Mellitus				1.000	Fisher's Exact
Absent	387 (92.36%)	59 (92.19%)	446		
Present	32 (7.64%)	5 (7.81%)	37		
Ischemic heart disease				0.024	Chi-square
Absent	355 (84.73%)	47 (73.44%)	402		
Present	64 (15.27%)	17 (26.56%)	81		

Timing					
	Acute (within 14 days)	Delayed (more than 14 days)	Total	*p*-value	Statistical test
Age	52 (44–59)	55 (47–62)	54 (46–61)	0.016	Wilcoxon two-sample test
Rural/city					
City	105 (57.07%)	117 (39.13%)	222	< 0.001	Chi-square
Rural	79 (42.93%)	182 (60.87%)	261		

### 3.2 Primary outcome and main multivariable predictors

At admission, 87.6% of patients presented with functional independence (mRS 0–2), while 12.4% demonstrated moderate-to-severe disability (mRS 3–6). At discharge, the proportion of functionally independent patients remained high at 84.8%, with 15.2% experiencing unfavorable outcomes (mRS 3–6), and with only 6 mortality cases. A paired Wilcoxon signed-rank test was used to evaluate differences in modified Rankin Scale (mRS) scores between admission and discharge. No statistically significant change was observed (*p* > 0.05). Of the total cohort, 470 patients (97.3%) had 12-month follow-up, among them 415 patients (88.2%) had mRS 0–2. No significant difference in outcome distribution was observed between these subgroups.

Among the cardiovascular and behavioral variables examined, smoking and ischemic heart disease (IHD) emerged as the only systemic factors consistently associated with unfavorable neurological outcome (mRS 3–6) in the overall cohort.

Smoking was associated with a significantly higher proportion of patients experiencing poor functional outcome compared with non-smokers (25.0% vs. 14.9%, *p* = 0.039).

In contrast, other vascular risk factors—including arterial hypertension across all stages, diabetes mellitus, and acute kidney injury—were not independently associated with functional outcome after adjustment.

Traditional SAH severity scales, including Hunt–Hess, WFNS, and Fisher grades, were likewise not associated with functional outcome after treatment (Hunt–Hess: *p* = 0.547; WFNS: *p* = 0.253; Fisher: *p* = 0.729; Supplementary Table S1). In the multivariable logistic regression analysis for mRS 3–6 outcomes, the presence of complications was the most significant predictor of a poor functional outcome, with an adjusted odds ratio (aOR) of 8.68 (95% CI: 2.78–27.22, *p* < 0.001) (Table 3 represents only statistically significant variables, all variables are represented in Supplementary Table S2). Patients with ischemic heart disease also faced significantly higher odds of a poor outcome (aOR 3.07, 95% CI: 1.25–7.30, *p* = 0.012), while higher Glasgow Coma Scale scores served as a significant protective factor, reducing the odds of poor outcome by 36% for every 1-unit increase (aOR 0.64, 95% CI: 0.50–0.82, *p* < 0.001). Furthermore, right-sided lesions (aOR 0.38, 95% CI: 0.15–0.90, *p* = 0.032) and the specific coil-balloon-stent procedural combination (aOR 0.10, 95% CI: 0.00–0.85, *p* = 0.032) were both associated with significantly lower odds of mRS 3–6 compared to their respective references. Notably, smoking status, which was significant in the unadjusted model, lost its statistical significance after adjustment for other variables (aOR 1.01, *p* = 0.978).

**Table 3:** Multivariable logistic regression analysis of predictors of unfavorable functional outcome (mRS 3–6).

Exposure	Level	OR (95% CI) Unadj.	*p*-value	OR (95% CI) Adj.	*p*-value
Residence	City	Reference	—	Reference	—
	Rural	0.83 (0.49–1.41)	0.487	0.72 (0.36–1.42)	0.343
Age	Per 5 units	1.04 (0.93–1.17)	0.512	0.93 (0.79–1.09)	0.340
Sex	Female	Reference	—	Reference	—
	Male	1.57 (0.91–2.67)	0.099	1.83 (0.88–3.78)	0.104
Side of aneurysm	Left	Reference	—	Reference	—
	Mixed	0.63 (0.34–1.16)	0.143	0.74 (0.34–1.58)	0.433
	**Right**	**0.42** (**0.21–0.81)**	**0.012**	**0.38** (**0.15–0.90)**	**0**.**032**
Procedure	Coil	Reference	—	Reference	—
	Coil/balloon	0.52 (0.24–1.22)	0.128	0.64 (0.28–1.56)	0.313
	Coil/balloon/angio	1.60 (0.26–7.56)	0.579	1.03 (0.14–5.55)	0.976
	**Coil/balloon/stent**	**0.07** (**0.00–0.56)**	**0.007**	**0.10** (**0.00–0.85)**	**0**.**032**
	Coil/stent	0.14 (0.00–1.26)	0.089	0.23 (0.00–2.09)	0.229
	Coil/WEB SL	1.39 (0.01–28.20)	0.849	2.49 (0.01–64.50)	0.631
	Stent	1.14 (0.50–2.76)	0.755	0.52 (0.15–1.64)	0.264
	Stent/angioplasty	1.39 (0.01–28.20)	0.849	2.93 (0.02–79.23)	0.577
Complications	No	Reference	—	Reference	—
	**Yes**	**9.45** (**3.75–24.49)**	**<0.001**	**8.68** (**2.78–27.22)**	**<0**.**001**
Smoking	Non-smoker	Reference	—	Reference	—
	Smoker	**1.92** (**1.00–3.53)**	**0.041**	1.01 (0.37–2.49)	0.978
Ischemic heart disease	Absent	Reference	—	Reference	—
	**Present**	**2.01** (**1.06–3.66)**	**0.027**	**3.07** (**1.25–7.30)**	**0**.**012**
Glasgow Coma Scale	**Per 1 unit**	**0.72** (**0.59–0.88)**	**0.001**	**0.64** (**0.50–0.82)**	**<0**.**001**

Bold values indicate statistically significant associations (*p* < 0.05).

### 3.3 Geographic access and treatment timing

In stratified analyses, periprocedural complications remained the dominant predictor of unfavorable outcome (mRS 3–6) across all subgroups, including rural and urban patients (both *p* = 0.002) and women and men (*p* = 0.007 and *p* = 0.012, respectively). Right-sided aneurysm location retained a protective association in urban patients (adjusted OR 0.26, 95% CI 0.08–0.76; *p* = 0.019) and in men (adjusted OR 0.15, 95% CI 0.03–0.57; *p* = 0.004). Delayed treatment was associated with lower odds of disability among women (adjusted OR 0.44, 95% CI 0.21–0.94; *p* = 0.034), whereas smoking was associated with higher odds of disability among men (adjusted OR 3.84, 95% CI 1.52–10.40; *p* = 0.004). All subgroup-specific regression estimates, including non-significant and unstable associations, are provided in Supplementary Tables S3 and S4.

### 3.4 System-Level vs. anatomical determinants of outcome

Most aneurysms were small (<7 mm, 71.6%) and saccular (95.5%). Aneurysm size, morphology, neck width, and arterial territory were not associated with functional outcome (all *p* > 0.20). No individual arterial location (ACA, ACom, ICA, MCA, or posterior circulation) independently predicted outcome (Supplementary Table S4).

Treatment strategies differed between care pathways. Acute management was predominantly coil-based with or without balloon assistance, whereas delayed treatment pathway (non-emergent) interventions more frequently employed stent-based or reconstructive approaches (*p* = 0.005). Within the delayed treatment pathway subgroup, procedural strategy was associated with outcome: stent-only treatment was independently associated with reduced disability (adjusted OR 0.13; *p* = 0.017), and combined coil/balloon/stent techniques showed an even stronger association with favorable outcome (adjusted OR 0.07; *p* = 0.024). Across all analyses, periprocedural complications were the strongest predictor of poor outcome (adjusted OR 8.68, 95% CI 2.78–27.22; *p* < 0.001) (Supplementary Table S5).

Procedure- or outcome-related complications occurred in 4.1% of patients, most commonly ischemic stroke (1.9%), vasospasm (1.6%), and hemorrhagic complications (0.6%). Overall mortality was low (1.2%). Fatal outcomes occurred in both acute and delayed groups and were predominantly observed in patients presenting with severe neurological impairment at admission. Non-vascular causes of death included urosepsis, thromboembolism, cerebral edema, and eclampsia. No postoperative complications were recorded in 95.9% of patients.

Angiographic recanalization was identified in 12.0% of aneurysms, and re-treatment was performed in 13.0% of cases, primarily for incomplete occlusion or stent-related issues. Functional independence (mRS 0–2) did not differ significantly between patients with and without recanalization (87.6% vs. 82.8%; *p* = 0.350). Smoking was the only independent predictor of recanalization, conferring more than a twofold increase in odds after adjustment for demographic, clinical, anatomical, and procedural factors (Supplementary Table S6).

In the final multivariable model, four factors remained independently associated with outcome: periprocedural complications (adjusted OR 8.68; *p* < 0.001), ischemic heart disease (adjusted OR 3.07; *p* = 0.012), admission Glasgow Coma Scale score (adjusted OR 0.64; *p* < 0.001), and right-sided aneurysm location (adjusted OR 0.38; *p* = 0.032).

### 3.5 Referral network and national care delivery

Kazakhstan's neurovascular care is organized around a centralized referral model, with two major tertiary cerebrovascular centers—one in Almaty and one in Astana—serving as the principal national referral hubs for complex aneurysm and neuroendovascular care. Both centers are considered high-volume, high-technology institutions equipped with advanced neurointerventional infrastructure and multidisciplinary expertise, receiving referrals from multiple regions across the country. Patients with aneurysmal subarachnoid hemorrhage requiring neurovascular intervention are frequently transferred from regional hospitals to these centers for definitive treatment, reflecting a hub-based model of care. This structure differs from many higher-income settings where neurovascular services may be distributed across broader networks of comprehensive centers and should be considered when interpreting the external validity of our findings.

**Figure 1:**
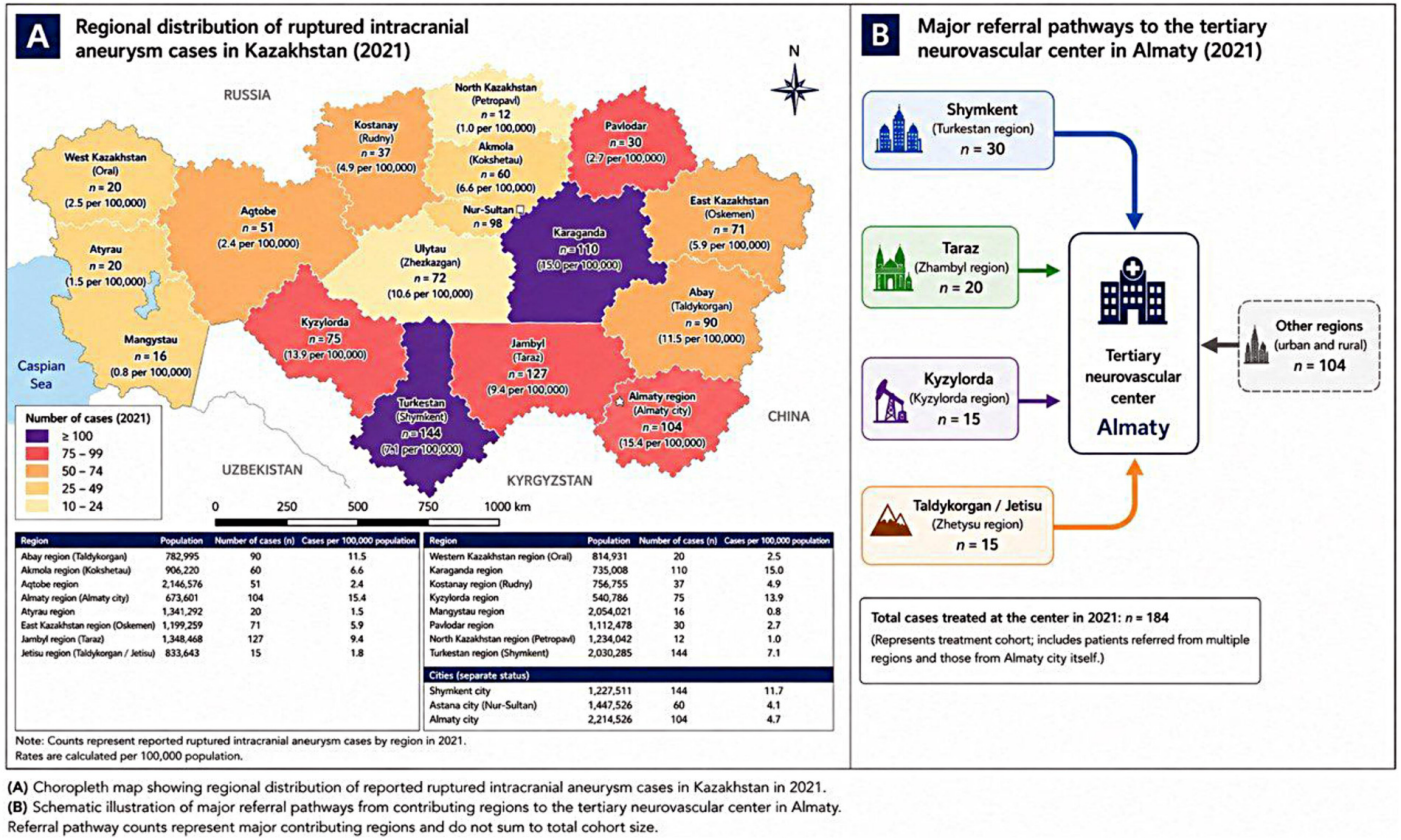
Geographic distribution of ruptured aneurysm cases and referral pathways within Kazakhstan (2021 representative year). The map illustrates the geographic distribution of reported ruptured intracranial aneurysm cases across Kazakhstan during a representative year. Arrows indicate referral flows from major contributing regions to the tertiary neurovascular center in Almaty. **(A)** Choropleth map showing the regional distribution of reported ruptured intracranial aneurysm cases in Kazakhstan during 2021. Colors indicate the number of reported cases by region, and incidence rates are presented per 100,000 population. **(B)** Schematic illustration of the major referral pathways from contributing regions to the tertiary neurovascular center in Almaty. The diagram highlights the principal referral hubs and the total number of patients treated at the tertiary center during the representative year. Referral mapping is presented for contextualization of healthcare access and was not included in regression analyses.

Referral mapping demonstrated that patients originated from multiple regions across Kazakhstan, illustrating reliance on a centralized neurovascular hub. Figure 1 illustrates national referral pathways to the tertiary neurovascular center using 2021 as a representative year. Of the 184 treated cases represented in the 2021 referral map, 104 originated from the Almaty metropolitan region, while the remainder were referred from other major contributing regions, including Shymkent (*n* = 30), Taraz (*n* = 20), Kyzylorda (*n* = 15), and Taldykorgan (*n* = 15).

The map illustrates the geographic distribution of reported ruptured intracranial aneurysm cases across Kazakhstan during a representative year. Arrows indicate referral flows from major contributing regions to the tertiary neurovascular center in Almaty.

**Figure 2:**
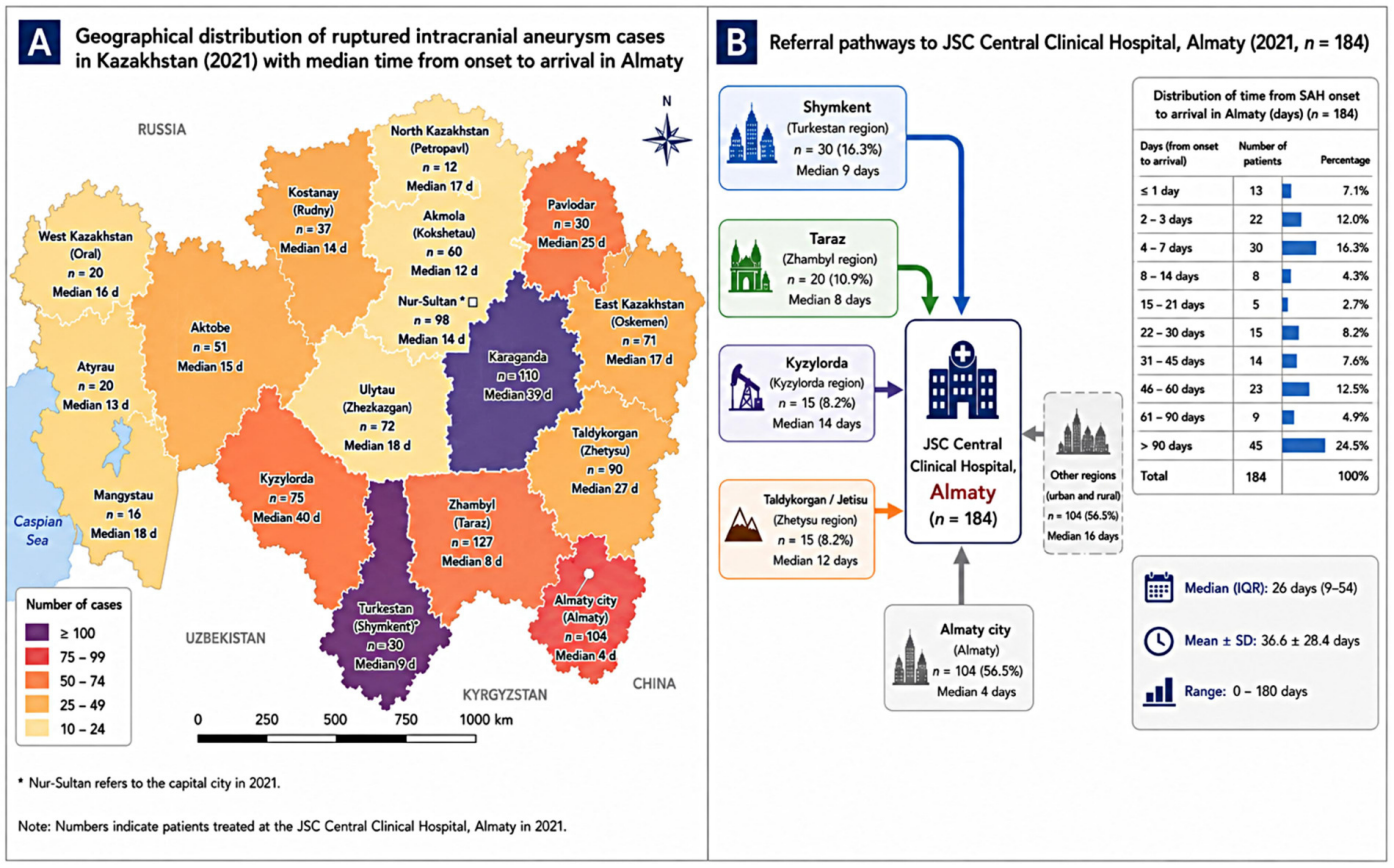
Geographical distribution of ruptured intracranial aneurysm cases in Kazakhstan and referral pathways to the tertiary neurovascular center in Almaty (2021). **(A)** Choropleth map showing the geographical distribution of patients with ruptured intracranial aneurysms treated at JSC Central Clinical Hospital, Almaty, in 2021. Colors indicate the number of treated cases by region. Median time from symptom onset to arrival at the tertiary center is displayed for each region. *Nur-Sultan refers to the capital city in 2021.* **(B)** Schematic representation of referral pathways to JSC Central Clinical Hospital, Almaty. Arrows indicate patient transfer from the major contributing regions and Almaty city to the tertiary neurovascular center. The histogram summarizes the distribution of time from SAH onset to hospital arrival, and the accompanying box reports the median (IQR), mean ± SD, and range of referral times. Numbers indicate patients treated at JSC Central Clinical Hospital, Almaty, in 2021. Referral mapping is presented for contextualization of healthcare access and was not included in regression analyses.

To further characterize referral dynamics and treatment accessibility, we analyzed time from SAH onset to arrival at the tertiary neurovascular center in a representative national referral cohort from 2021 (Figure 2). The median interval from SAH onset to arrival at the tertiary neurovascular center in Almaty was 26 days (IQR 9–54). A substantial proportion of patients were transferred during subacute or delayed stages after initial stabilization at regional hospitals.

Only 13 patients (7.1%) arrived within the first 24 h after hemorrhage, whereas nearly one-quarter of patients (24.5%) presented more than 90 days after onset. The median interval from SAH onset to arrival at JSC Central Clinical Hospital was 26 days (IQR 9–54).

## 4 Discussion

This study demonstrates that functional recovery after aneurysmal subarachnoid hemorrhage within a centralized upper-middle-income neurovascular system is influenced more strongly by baseline neurological status, systemic vascular comorbidity, and procedural safety than by aneurysm anatomy. Higher admission GCS scores were independently protective, whereas ischemic heart disease and periprocedural complications were associated with unfavorable outcome (mRS 3–6). In contrast, aneurysm morphology and most anatomical characteristics were not independently associated with long-term disability. These findings suggest that, once patients successfully access definitive treatment for the ruptured aneurysm, recovery may depend more on patient-level health status and treatment-related factors than on lesion-specific complexity alone.

### 4.1 Modifiable risk factors as determinants of disability

Smoking likely contributes to poorer outcome through endothelial dysfunction, impaired vascular healing, increased inflammatory activity, and higher rates of aneurysm instability and recanalization (14). Similarly, ischemic heart disease may reflect generalized systemic vascular dysfunction and reduced physiological reserve during acute neurocritical illness (15). These findings reinforce the importance of cardiovascular optimization and risk-factor modification in patients with aneurysmal SAH. These conditions are preventable and amenable to public-health intervention, suggesting that reductions in post-SAH disability may be achievable through strengthened cardiovascular prevention strategies.

The independent association between smoking and aneurysm recanalization further emphasizes the role of behavioral risk in long-term treatment durability, positioning lifestyle modification as a relevant component of neurovascular care beyond the acute phase.

### 4.2 Interpretation within global neurosurgery

These findings contribute to the evolving global neurosurgery perspective that disparities in neurosurgical outcomes often arise from system organization rather than purely clinical factors. In transitional healthcare systems, inequity may manifest through differences in referral pathways, transport logistics, and stabilization capacity rather than in-hospital treatment quality. The median interval of 26 days from SAH onset to arrival at the tertiary center highlights the substantial referral and transfer delays present within the centralized neurovascular system.

The observed preservation of functional outcomes despite geographic variation highlights the potential effectiveness of centralized referral models when supported by coordinated infrastructure and specialized expertise. Our findings align with prior international studies demonstrating that neurological severity and systemic comorbidity are major determinants of recovery after aneurysmal SAH (16, 17]. Similar associations between smoking, cardiovascular disease, and poorer functional outcome have been reported in both surgical and endovascular cohorts (18–20). In contrast to several earlier studies identifying aneurysm size and morphology as prognostic markers (21, 22), anatomical variables were not independently associated with outcome in the present cohort. This discrepancy may reflect improvements in contemporary endovascular techniques, which have reduced anatomy-driven procedural risk, while shifting the relative importance toward systemic health status, perioperative complications, and healthcare access.

### 4.3 Equity, access, and regional care delivery

Rural residence was associated with delayed access to definitive treatment, reflecting transfer distance and limited regional neurointerventional capacity. Nevertheless, rural residence itself was not independently associated with worse functional outcome among treated patients. This observation suggests that centralized tertiary neurovascular systems may partially buffer geographic disparities once specialized care is successfully reached (23). However, interpretation requires caution because the study cohort inherently excludes patients who died before transfer or were unable to access tertiary treatment.

### 4.4 Implications for public health policy

These findings have several practical implications for healthcare systems in upper-middle-income settings:

1. Strengthening peri-procedural safety protocols may substantially improve functional outcomes.
2. Aggressive cardiovascular risk-factor management, particularly smoking cessation and optimization of ischemic heart disease, should be integrated into neurovascular care pathways.
3. Coordinated transfer systems and expansion of regional stroke infrastructure may reduce treatment delays while preserving the advantages of centralized expertise.

## 5 Limitations

This study has several limitations. First, the retrospective single-center design limits causal inference and generalizability. Second, the cohort represents a treatment-only tertiary referral population and is subject to substantial survivorship and selection bias because patients who died before transfer or were not eligible for intervention were not captured. Third, socioeconomic variables such as income, education, and insurance status were unavailable. Fourth, mixed 6- and 12-month follow-up intervals may introduce variability in functional outcome assessment. Fifth, some subgroup analyses were limited by small sample sizes and should be considered exploratory. Finally, external validation in other upper-middle-income neurovascular systems is required before broader generalization of these findings. Future studies with standardized follow-up intervals are needed to confirm the durability of these findings.

## 6 Conclusion

This study demonstrates that functional outcomes after aneurysmal subarachnoid hemorrhage within a centralized upper-middle-income neurovascular system are shaped primarily by baseline neurological status, systemic vascular comorbidity, and procedural safety rather than aneurysm anatomy. Geographic variation influenced treatment pathways, with rural patients more frequently undergoing stabilization and delayed intervention; however, comparable functional outcomes across residence groups suggest that coordinated referral and centralized referral networks may help preserve functional outcomes among patients who successfully reach specialized care despite disparities in access.

Modifiable cardiovascular risk factors—particularly smoking and ischemic heart disease—emerged as consistent determinants of disability and treatment durability, highlighting the importance of integrating vascular prevention into neurovascular care pathways. The dominant impact of periprocedural complications further underscores the role of quality and safety frameworks in improving outcomes within specialized centers.

Collectively, these findings indicate that improving population-level recovery after aneurysmal SAH requires strategies extending beyond technical treatment, including strengthened referral networks, equitable access to specialized services, and systematic cardiovascular risk reduction. These findings suggest that among patients who successfully reach specialized care, centralized referral systems may help preserve functional outcomes despite geographic disparities in access.

## Data Availability

The original contributions presented in the study are included in the article/Supplementary Material, further inquiries can be directed to the corresponding author.
