# Supplementary material for: Access to care, treatment pathways, and outcomes of endovascular treatment for aneurysmal subarachnoid hemorrhage in Kazakhstan: a retrospective cohort study
Source: Front Radiol. 2026 Jun 26;6:1845453. doi: 10.3389/fradi.2026.1845453 (PMC13350253; doi:10.3389/fradi.2026.1845453)
Supplement: Supplementary file 1 [file Datasheet1.docx]

| **Supplementary Table** 1**. Clinical severity scales at admission** | | | | | |
| --- | --- | --- | --- | --- | --- |
| **Hunt Hess grading** | |  |  | 546 | Fisher's Exact |
| **1** | 312 (82.11%) | 33 (80.49%) | 345 |  |  |
| **2** | 27 (7.11%) | 2 (4.88%) | 29 |  |  |
| **3** | 23 (6.05%) | 2 (4.88%) | 25 |  |  |
| **4** | 18 (4.74%) | 4 (9.76%) | 22 |  |  |
| **WFNS scale** |  |  |  | 253 | Fisher's Exact |
| **1** | 310 (81.58%) | 33 (80.49%) | 343 |  |  |
| **2** | 26 (6.84%) | 2 (4.88%) | 28 |  |  |
| **3** | 25 (6.58%) | 1 (2.44%) | 26 |  |  |
| **4** | 19 (5.00%) | 5 (12.20%) | 24 |  |  |
| **Fisher scale** |  |  |  | 729 | Fisher's Exact |
| **1** | 315 (82.89%) | 34 (82.93%) | 349 |  |  |
| **2** | 12 (3.16%) | 0 (0.00%) | 12 |  |  |
| **3** | 12 (3.16%) | 1 (2.44%) | 13 |  |  |
| **4** | 41 (10.79%) | 6 (14.63%) | 47 |  |  |

| **Supplementary Table 2. Multivariable logistic regression analysis of predictors of unfavorable functional outcome (mRS 3–6)** | | | | | |
| --- | --- | --- | --- | --- | --- |
| **Exposure** | **Level** | **OR (95% CI) Unadj.** | **p-value** | **OR (95% CI) Adj.** | **p-value** |
| **Residence** | City | Reference | — | Reference | — |
|  | Rural | 0.83 (0.49–1.41) | 487 | 0.72 (0.36–1.42) | 343 |
| **Age** | Per 5 units | 1.04 (0.93–1.17) | 512 | 0.93 (0.79–1.09) | 0.340 |
| **Sex** | Female | Reference | — | Reference | — |
|  | Male | 1.57 (0.91–2.67) | 99 | 1.83 (0.88–3.78) | 104 |
| **Occupation** | High stress | Reference | — | Reference | — |
|  | Low stress | 1.15 (0.68–1.97) | 611 | 1.28 (0.65–2.58) | 487 |
| **Side of lesion** | Left | Reference | — | Reference | — |
|  | Mixed | 0.63 (0.34–1.16) | 143 | 0.74 (0.34–1.58) | 433 |
|  | **Right** | **0.42 (0.21–0.81)** | **12** | **0.38 (0.15–0.90)** | **32** |
| **Artery involved** | ACA | Reference | — | Reference | — |
|  | Acom | 1.10 (0.26–7.59) | 909 | 0.85 (0.17–6.27) | 848 |
|  | AICA | —* | 992 | —* | — |
|  | BA | 0.86 (0.04–10.64) | 907 | —* | 987 |
|  | ICA | 1.12 (0.28–7.41) | 0.89 | 0.73 (0.16–5.37) | 719 |
|  | MCA | 0.69 (0.16–4.75) | 0.650 | 0.41 (0.08–3.13) | 321 |
|  | Multi | 0.54 (0.10–4.05) | 486 | 0.51 (0.09–4.14) | 476 |
|  | PCA | —* | 992 | —* | 995 |
|  | Pcom | —* | 992 | —* | 995 |
|  | PICA | 6.00 (0.19–206.71) | 265 | 1.92 (0.03–106.52) | 742 |
| **Vessels involved** | Multi | Reference | — | Reference | — |
|  | Single | 2.01 (0.94–4.98) | 95 | 1.29 (0.56–3.34) | 572 |
| **SAH type** | Acute | Reference | — | Reference | — |
|  | Delayed | 0.61 (0.36–1.04) | 69 | 0.68 (0.32–1.47) | 323 |
| **Procedure** | Coil | Reference | — | Reference | — |
|  | Coil / balloon | 0.52 (0.24–1.22) | 128 | 0.64 (0.28–1.56) | 313 |
|  | Coil / balloon / angio | 1.60 (0.26–7.56) | 579 | 1.03 (0.14–5.55) | 976 |
|  | **Coil / balloon / stent** | **0.07 (0.00–0.56)** | **7** | **0.10 (0.00–0.85)** | **32** |
|  | Coil / stent | 0.14 (0.00–1.26) | 89 | 0.23 (0.00–2.09) | 229 |
|  | Coil / WEB SL | 1.39 (0.01–28.20) | 849 | 2.49 (0.01–64.50) | 631 |
|  | Stent | 1.14 (0.50–2.76) | 755 | 0.52 (0.15–1.64) | 264 |
|  | Stent / angioplasty | 1.39 (0.01–28.20) | 849 | 2.93 (0.02–79.23) | 577 |
| **Broad-neck** | No | Reference | — | Reference | — |
|  | Yes | 0.72 (0.39–1.30) | 292 | 1.04 (0.46–2.25) | 921 |
| **Aneurysm Size** | Giant | Reference | — | Reference | — |
|  | Large | 0.95 (0.16–5.69) | 953 | 0.41 (0.04–3.26) | 406 |
|  | Medium | 0.63 (0.17–3.09) | 528 | 0.37 (0.08–2.04) | 213 |
|  | Small | 1.06 (0.34–4.61) | 933 | 0.61 (0.17–2.98) | 0.490 |
| **Shape** | Blister | Reference | — | Reference | — |
|  | Dissectant | —* | 986 | 0.36 (0.00–8.55) | 552 |
|  | Fenestrated | —* | 992 | 0.29 (0.00–12.52) | 528 |
|  | Fusiform | 0.44 (0.02–5.56) | 534 | 0.20 (0.00–3.39) | 287 |
|  | Saccular | 0.53 (0.13–3.64) | 0.440 | 0.43 (0.09–2.73) | 336 |
| **Complications** | No | Reference | — | Reference | — |
|  | **Yes** | **9.45 (3.75–24.49)** | **<0.001** | **8.68 (2.78–27.22)** | **<0.001** |
| **Smoking** | Non-smoker | Reference | — | Reference | — |
|  | Smoker | **1.92 (1.00–3.53)** | **41** | 1.01 (0.37–2.49) | 978 |
| **Hypertension** | Absent | Reference | — | Reference | — |
|  | Stage 1 | 1.70 (0.55–5.60) | 363 | 1.39 (0.29–6.29) | 666 |
|  | Stage 2 | 0.47 (0.11–1.78) | 272 | 0.26 (0.03–1.47) | 153 |
|  | Stage 3 | 0.83 (0.35–2.30) | 693 | 0.74 (0.25–2.56) | 0.610 |
| **Diabetes** | Absent | Reference | — | Reference | — |
|  | Present | 1.02 (0.34–2.52) | 961 | 1.16 (0.29–3.64) | 815 |
| **Ischemic heart** | Absent | Reference | — | Reference | — |
|  | **Present** | **2.01 (1.06–3.66)** | **27** | **3.07 (1.25–7.30)** | **12** |
| **Recanalized** | Absent | Reference | — | Reference | — |
|  | Present | 1.46 (0.69–2.89) | 293 | 0.83 (0.22–2.38) | 755 |
| **Hunt-Hess grading** | Per 1 unit | 1.27 (0.89–1.74) | 152 | 1.06 (0.70–1.53) | 763 |
| **Glasgow Coma Scale** | **Per 1 unit** | **0.72 (0.59–0.88)** | **1** | **0.64 (0.50–0.82)** | **<0.001** |

| **Supplementary Table S**3**.** Multivariable logistic regression analysis of predictors of unfavorable functional outcome (mRS 3–6), stratified by sex | | | | | |
| --- | --- | --- | --- | --- | --- |
| **Exposure** | **Level** | **OR (95% CI) Female** | **p-value** | **OR (95% CI) Male** | **p-value** |
| **Residence** | City | Reference | — | Reference | — |
|  | Rural | 0.94 (0.46–1.93) | 873 | 0.70 (0.30–1.65) | 417 |
| **Age** | Per 5 units | 1.13 (0.95–1.36) | 162 | 0.84 (0.68–1.03) | 91 |
| **Occupation** | High stress | Reference | — | Reference | — |
|  | Low stress | 1.60 (0.78–3.41) | 206 | 0.90 (0.38–2.10) | 804 |
| **Side of lesion** | Left | Reference | — | Reference | — |
|  | Mixed | 0.49 (0.19–1.18) | 114 | 0.67 (0.26–1.72) | 405 |
|  | **Right** | 0.77 (0.34–1.77) | 544 | **0.15 (0.03–0.57)** | **4** |
| **Artery involved** | ACA | Reference | — | Reference | — |
|  | Acom | 1.29 (0.08–203.63) | 878 | 0.80 (0.15–5.22) | 797 |
|  | AICA | 5.65 (0.02–1693.48) | 479 | —* | — |
|  | BA | 5.69 (0.02–1382.42) | 449 | 0.54 (0.03–6.39) | 624 |
|  | ICA | 3.23 (0.28–475.90) | 407 | 0.60 (0.12–3.94) | 564 |
|  | MCA | 1.75 (0.14–264.49) | 719 | 0.42 (0.07–2.92) | 354 |
|  | **Multi** | 3.02 (0.22–464.67) | 459 | **0.05 (0.00–0.83)** | **36** |
|  | PCA | 4.02 (0.02–1043.02) | 549 | —* | — |
|  | Pcom | 5.01 (0.02–1591.39) | 514 | —* | — |
|  | PICA | 15.87 (0.27–7159.09) | 196 | 2.28 (0.01–71.80) | 672 |
| **Vessels involved** | Multi | Reference | — | Reference | — |
|  | **Single** | 1.01 (0.42–2.71) | 991 | **5.37 (1.19–51.89)** | **26** |
| **SAH type** | Acute | Reference | — | Reference | — |
|  | **Delayed** | **0.44 (0.21–0.94)** | **34** | 0.69 (0.28–1.66) | 0.400 |
| **Procedure** | Coil | Reference | — | Reference | — |
|  | **Coil / balloon** | 1.17 (0.33–6.13) | 824 | **0.23 (0.07–0.77)** | **18** |
|  | Coil / balloon / angioplasty | 1.32 (0.01–24.84) | 872 | 2.62 (0.29–32.68) | 386 |
|  | Coil / balloon / stent | 0.19 (0.00–2.63) | 237 | 0.11 (0.00–1.33) | 89 |
|  | Coil / stent | 0.39 (0.00–5.54) | 528 | 0.10 (0.00–1.61) | 113 |
|  | Coil / WEB SL | 1.94 (0.01–95.62) | 0.750 | —* | — |
|  | Stent | 2.07 (0.56–11.23) | 296 | 0.35 (0.09–1.26) | 106 |
|  | Stent / angioplasty | 7.32 (0.04–261.21) | 350 | —* | — |
| **Broad-neck** | No | Reference | — | Reference | — |
|  | Yes | 0.89 (0.38–2.00) | 781 | 0.47 (0.16–1.25) | 132 |
| **Aneurysm Size** | Giant | Reference | — | Reference | — |
|  | Large | 1.09 (0.08–11.08) | 944 | 0.76 (0.07–11.11) | 829 |
|  | Medium | 1.01 (0.21–6.81) | 0.990 | 0.23 (0.01–3.43) | 261 |
|  | Small | 1.00 (0.25–6.26) | 999 | 0.75 (0.12–8.11) | 782 |
| **Shape** | Blister | Reference | — | Reference | — |
|  | Dissectant | 0.25 (0.00–6.84) | 431 | 0.84 (0.00–242.19) | 941 |
|  | Fenestrated | —* | — | 0.38 (0.00–138.34) | 0.71 |
|  | Fusiform | 0.06 (0.00–1.12) | 0.060 | 2.41 (0.06–511.13) | 655 |
|  | Saccular | 0.20 (0.04–1.32) | 0.090 | 1.62 (0.11–239.59) | 0.760 |
| **Complications** | No | Reference | — | Reference | — |
|  | **Yes** | **6.93 (1.74–28.85)** | **7** | **5.91 (1.47–27.82)** | **12** |
| **Smoking** | Non-smoker | Reference | — | Reference | — |
|  | **Smoker** | **0.12 (0.00–0.94)** | **42** | **3.84 (1.52–10.40)** | **4** |
| **Hypertension** | Absent | Reference | — | Reference | — |
|  | Stage 1 | 4.70 (0.73–52.33) | 105 | 0.62 (0.13–2.84) | 537 |
|  | **Stage 2** | 1.69 (0.28–18.16) | 587 | **0.06 (0.00–0.69)** | **0.020** |
|  | Stage 3 | 1.31 (0.28–12.67) | 762 | 0.54 (0.17–1.92) | 324 |
| **Diabetes** | Absent | Reference | — | Reference | — |
|  | Present | 0.63 (0.12–2.17) | 494 | 1.24 (0.26–4.98) | 772 |
| **Ischemic heart** | Absent | Reference | — | Reference | — |
|  | **Present** | **2.43 (1.00–5.64)** | **49** | 1.31 (0.40–3.90) | 643 |
| **Recanalized** | Per 1 unit(s) | 1.52 (0.50–4.01) | 433 | 1.51 (0.50–4.20) | 447 |

| **Supplementary Table S4.** Multivariable logistic regression analysis of predictors of unfavorable functional outcome (mRS 3–6), stratified by residence | | | | | |
| --- | --- | --- | --- | --- | --- |
| **Exposure** | **Level** | **OR (95% CI) Rural** | **p-value adj.** | **OR (95% CI) City** | **p-value adj.** |
| **Age** | Per 5 unit(s) | 1.00 (0.84–1.21) | 976 | 1.03 (0.85–1.26) | 748 |
| **Sex** | Female | Reference | — | Reference | — |
|  | Male | 0.97 (0.39–2.35) | 954 | 2.02 (0.82–4.88) | 119 |
| **Occupation** | high stress | Reference | — | Reference | — |
|  | low stress | 0.78 (0.36–1.71) | 535 | 2.16 (0.96–5.20) | 0.070 |
| **Side of lesion** | Left | Reference | — | Reference | — |
|  | Mixed | 0.55 (0.20–1.43) | 228 | 0.53 (0.20–1.32) | 177 |
|  | **Right** | 0.57 (0.21–1.44) | 237 | **0.26 (0.08–0.76)** | **19** |
| **Artery involved** | ACA | Reference | — | Reference | — |
|  | Acom | 1.02 (0.13–21.77) | 988 | 0.79 (0.09–17.33) | 0.850 |
|  | AICA | —* | 995 | —* | — |
|  | BA | —* | 992 | 0.58 (0.02–20.51) | 739 |
|  | ICA | 1.19 (0.17–24.80) | 879 | 1.01 (0.13–21.24) | 991 |
|  | MCA | 0.41 (0.05–9.00) | 467 | 0.90 (0.11–19.42) | 932 |
|  | Multi | 0.37 (0.03–9.17) | 459 | 0.67 (0.06–15.67) | 754 |
|  | PCA | —* | 995 | —* | — |
|  | Pcom | —* | 995 | —* | — |
|  | PICA | 7.49 (0.17–441.28) | 275 | —* | — |
| **Vessels involved** | Multi | Reference | — | Reference | — |
|  | Single | 2.11 (0.67–9.35) | 251 | 1.60 (0.56–5.76) | 415 |
| **SAH type** | Acute | Reference | — | Reference | — |
|  | Delayed | 0.63 (0.28–1.46) | 271 | 0.60 (0.26–1.34) | 216 |
| **Procedure** | Coil | Reference | — | Reference | — |
|  | Coil / balloon | 0.88 (0.28–3.29) | 839 | 0.41 (0.13–1.36) | 137 |
|  | Coil / balloon / angioplasty | 5.17 (0.51–51.67) | 156 | 0.27 (0.00–3.43) | 354 |
|  | Coil / balloon / stent | 0.21 (0.00–2.27) | 0.230 | 0.12 (0.00–1.29) | 87 |
|  | Coil / stent | 0.55 (0.00–6.66) | 681 | 0.18 (0.00–2.26) | 209 |
|  | Coil / WEB SL | 1.15 (0.01–41.66) | 943 | —* | — |
|  | Stent | 1.99 (0.61–7.57) | 0.260 | 0.77 (0.22–2.88) | 692 |
|  | Stent / angioplasty | 2.57 (0.02–74.17) | 625 | —* | — |
| **Broad-neck** | no | Reference | — | Reference | — |
|  | yes | 0.88 (0.33–2.17) | 787 | 0.82 (0.31–2.05) | 683 |
| **Aneurysm Size** | giant | Reference | — | Reference | — |
|  | large | 0.21 (0.01–2.05) | 214 | —* | 986 |
|  | medium | 0.51 (0.10–3.05) | 436 | —* | 988 |
|  | small | 0.65 (0.17–3.22) | 552 | —* | 987 |
| **Shape** | blister | Reference | — | Reference | — |
|  | dissectant | —* | 995 | —* | 993 |
|  | fenestrated | —* | — | —* | 995 |
|  | fusiform | —* | 989 | 0.64 (0.02–23.34) | 0.790 |
|  | saccular | 0.21 (0.02–5.31) | 252 | 0.72 (0.09–15.66) | 784 |
| **Complications** | No | Reference | — | Reference | — |
|  | **Yes** | **10.04 (2.27–48.50)** | **2** | **11.17 (2.55–56.98)** | **2** |
| **Smoking** | Non-smoker | Reference | — | Reference | — |
|  | Smoker | 2.32 (0.88–6.01) | 84 | 0.91 (0.25–2.84) | 874 |
| **Hypertension** | Absent | Reference | — | Reference | — |
|  | Stage 1 | 2.18 (0.36–18.07) | 416 | 1.25 (0.23–6.74) | 793 |
|  | Stage 2 | 0.26 (0.01–3.06) | 296 | 0.75 (0.12–4.28) | 746 |
|  | Stage 3 | 0.95 (0.22–6.73) | 955 | 0.63 (0.18–2.62) | 488 |
| **Diabetes** | Absent | Reference | — | Reference | — |
|  | Present | 1.05 (0.27–3.30) | 0.940 | 0.51 (0.03–3.12) | 544 |
| **Ischemic heart** | Absent | Reference | — | Reference | — |
|  | Present | 1.69 (0.61–4.33) | 288 | 2.56 (0.91–6.95) | 67 |
| **Recanalized** | Per 1 unit(s) | 2.42 (0.90–6.05) | 66 | 0.63 (0.14–2.08) | 489 |

| **Supplementary Table S5. Multivariable logistic regression analysis of predictors of unfavorable functional outcome (mRS 3–6), stratified by treatment timing** | | | | | |
| --- | --- | --- | --- | --- | --- |
| **Exposure** | **Level** | **OR (95% CI) Acute** | **p-value** | **OR (95% CI) Delayed** | **p-value** |
| **Residence** | City | Reference | — | Reference | — |
|  | Rural | 0.77 (0.27–2.04) | 608 | 0.61 (0.23–1.65) | 321 |
| **Age** | Per 5 units | 0.92 (0.73–1.16) | 471 | 0.96 (0.76–1.23) | 752 |
| **Sex** | Female | Reference | — | Reference | — |
|  | Male | 1.61 (0.58–4.44) | 354 | 2.06 (0.71–5.98) | 182 |
| **Occupation** | High stress | Reference | — | Reference | — |
|  | Low stress | 0.74 (0.28–1.97) | 536 | 1.68 (0.64–4.86) | 298 |
| **Side of lesion** | Left | Reference | — | Reference | — |
|  | Mixed | 1.14 (0.39–3.43) | 813 | 0.46 (0.15–1.33) | 154 |
|  | **Right** | 0.55 (0.15–1.89) | 347 | **0.29 (0.07–0.98)** | **46** |
| **Artery involved** | ACA | Reference | — | Reference | — |
|  | Acom | 0.68 (0.05–17.34) | 774 | 0.95 (0.13–11.78) | 964 |
|  | BA | —* | 991 | 0.78 (0.00–26.65) | 898 |
|  | ICA | 0.58 (0.05–14.50) | 683 | 0.93 (0.14–11.48) | 949 |
|  | MCA | 0.16 (0.01–4.39) | 198 | 0.91 (0.13–11.02) | 929 |
|  | Multi | 0.29 (0.02–8.21) | 392 | 0.72 (0.08–9.94) | 784 |
|  | Pcom | —* | 995 | 13.88 (0.05–2011.84) | 296 |
|  | PICA | —* | 995 | 2.33 (0.01–101.46) | 676 |
| **Vessels involved** | Multi | Reference | — | Reference | — |
|  | Single | 0.90 (0.30–3.13) | 857 | 1.82 (0.54–9.44) | 363 |
| **Procedure** | Coil | Reference | — | Reference | — |
|  | Coil / balloon | 1.10 (0.31–4.74) | 886 | 0.31 (0.08–1.21) | 89 |
|  | Coil / balloon / angioplasty | 1.77 (0.16–15.10) | 606 | 1.35 (0.01–37.26) | 871 |
|  | Coil / balloon / stent | —* | 995 | **0.07 (0.00–0.75)** | **24** |
|  | Coil / stent | —* | 995 | 0.16 (0.00–1.84) | 0.160 |
|  | Coil / WEB SL | —* | — | 1.00 (0.01–39.63) | 999 |
|  | Stent | 3.48 (0.35–34.05) | 275 | **0.13 (0.02–0.70)** | **17** |
|  | Stent / angioplasty | —* | — | 1.720 (0.01–58.75) | 781 |
| **Broad-neck** | No | Reference | — | Reference | — |
|  | Yes | 1.40 (0.47–4.09) | 534 | 0.810 (0.23–2.59) | 732 |
| **Aneurysm Size** | Giant | Reference | — | Reference | — |
|  | Large | 0.90 (0.05–12.85) | 0.940 | 0.580 (0.00–114.01) | 792 |
|  | Medium | 0.220 (0.03–1.76) | 141 | 1.230 (0.09–177.16) | 896 |
|  | Small | 0.36 (0.06–2.37) | 253 | 1.58 (0.17–212.14) | 746 |
| **Shape** | Blister | Reference | — | Reference | — |
|  | Dissectant | —* | — | 1.18 (0.00–330.97) | 945 |
|  | Fenestrated | —* | — | 0.24 (0.00–92.03) | 589 |
|  | Fusiform | —* | 992 | 0.53 (0.00–131.88) | 778 |
|  | Saccular | 0.41 (0.05–4.65) | 423 | 0.88 (0.06–139.01) | 941 |
| **Complications** | No | Reference | — | Reference | — |
|  | **Yes** | **6.71 (1.60–29.69)** | **9** | **8.69 (1.11–61.13)** | **41** |
| **Smoking** | Non-smoker | Reference | — | Reference | — |
|  | Smoker | 1.00 (0.22–3.80) | 999 | 0.87 (0.20–3.01) | 839 |
| **Hypertension** | Absent | Reference | — | Reference | — |
|  | Stage 1 | 3.24 (0.43–25.71) | 248 | 0.73 (0.06–7.15) | 782 |
|  | Stage 2 | 0.27 (0.01–3.19) | 343 | 0.42 (0.03–3.86) | 435 |
|  | Stage 3 | 0.75 (0.17–4.06) | 714 | 0.56 (0.12–3.66) | 508 |
| **Diabetes Mellitus** | Absent | Reference | — | Reference | — |
|  | Present | 0.35 (0.02–2.69) | 393 | 2.40 (0.49–9.66) | 259 |
| **Ischemic heart** | Absent | Reference | — | Reference | — |
|  | **Present** | 2.73 (0.70–10.36) | 0.140 | **3.53 (1.06–10.96)** | **0.040** |
| **Recanalized** | Absent | Reference | — | Reference | — |
|  | Present | 0.45 (0.02–3.24) | 505 | 1.19 (0.28–3.87) | 795 |
| **Hunt-Hess grading** | Per 1 unit | 1.43 (0.86–2.30) | 0.150 | 0.69 (0.21–1.35) | 321 |
| **Glasgow Coma Scale** | **Per 1 unit** | **0.70 (0.52–0.92)** | **14** | 0.55 (0.13–6.63) | 439 |

| **Supplementary Table S6.** Logistic regression analysis of predictors of aneurysm recanalization | | | | | |
| --- | --- | --- | --- | --- | --- |
| **Exposure** | **Level** | **OR (95% CI) Unadj.** | **p-value** | **OR (95% CI) Adj.** | **p-value** |
| **Residence** | City | Reference | — | Reference | — |
|  | Rural | 1.00 (0.59–1.70) | 991 | 0.97 (0.56–1.69) | 924 |
| **Age** | Per 5 units | 0.95 (0.85–1.06) | 359 | 0.95 (0.84–1.09) | 474 |
| **Sex** | Female | Reference | — | Reference | — |
|  | Male | 1.39 (0.80–2.38) | 232 | 0.99 (0.52–1.85) | 981 |
| **Occupation** | High stress | Reference | — | Reference | — |
|  | Low stress | 0.83 (0.49–1.41) | 487 | 0.82 (0.48–1.42) | 485 |
| **Side of lesion** | Left | Reference | — | Reference | — |
|  | Mixed | 1.14 (0.61–2.15) | 682 | 1.21 (0.63–2.31) | 569 |
|  | Right | 0.76 (0.38–1.50) | 427 | 0.78 (0.38–1.55) | 474 |
| **Artery involved** | ACA | Reference | — | Reference | — |
|  | Acom | 0.67 (0.18–3.26) | 579 | 0.60 (0.15–3.03) | 492 |
|  | AICA | —* | 992 | —* | 992 |
|  | BA | 0.52 (0.02–5.09) | 605 | 0.51 (0.02–5.27) | 597 |
|  | ICA | 0.52 (0.15–2.43) | 343 | 0.50 (0.13–2.44) | 332 |
|  | MCA | 0.39 (0.10–1.87) | 185 | 0.34 (0.08–1.73) | 0.150 |
|  | Multi | 0.72 (0.18–3.59) | 655 | 0.75 (0.18–3.93) | 709 |
|  | PCA | —* | 991 | —* | 991 |
|  | Pcom | —* | 992 | —* | 992 |
|  | PICA | —* | 989 | —* | 989 |
| **Vessels involved** | Multi | Reference | — | Reference | — |
|  | Single | 0.86 (0.46–1.73) | 662 | 0.80 (0.42–1.63) | 521 |
| **SAH type** | Acute | Reference | — | Reference | — |
|  | Delayed | 1.63 (0.93–2.99) | 97 | 1.59 (0.89–2.96) | 129 |
| **Procedure** | Coil | Reference | — | Reference | — |
|  | Coil / balloon | 0.54 (0.23–1.44) | 185 | 0.52 (0.21–1.43) | 179 |
|  | Coil / balloon / angio | 1.95 (0.25–10.71) | 464 | 1.61 (0.20–9.19) | 612 |
|  | Coil / balloon / stent | 1.41 (0.41–4.70) | 578 | 1.47 (0.42–5.06) | 541 |
|  | Coil / stent | 1.60 (0.31–6.86) | 543 | 1.32 (0.24–5.95) | 726 |
|  | Coil / WEB SL | —* | 985 | —* | 985 |
|  | Stent | 1.41 (0.58–3.83) | 467 | 1.27 (0.50–3.54) | 622 |
|  | Stent / angioplasty | —* | 988 | —* | 989 |
| **Broad-neck** | No | Reference | — | Reference | — |
|  | Yes | 0.71 (0.36–1.30) | 279 | 0.70 (0.35–1.32) | 283 |
| **Aneurysm Size** | Giant | Reference | — | Reference | — |
|  | Large | 1.50 (0.23–12.33) | 675 | 1.35 (0.20–11.31) | 756 |
|  | Medium | 1.58 (0.39–10.67) | 0.570 | 1.48 (0.35–10.17) | 629 |
|  | Small | 1.51 (0.42–9.67) | 585 | 1.40 (0.38–9.07) | 0.660 |
| **Shape** | Blister | Reference | — | Reference | — |
|  | Dissectant | —* | 986 | —* | 986 |
|  | Fenestrated | —* | 991 | —* | 991 |
|  | Fusiform | 0.44 (0.02–5.56) | 534 | 0.58 (0.02–7.78) | 689 |
|  | Saccular | 0.51 (0.12–3.50) | 413 | 0.62 (0.13–4.49) | 0.580 |
| **Complications** | No | Reference | — | Reference | — |
|  | Yes | 1.19 (0.27–3.66) | 791 | 0.86 (0.19–2.81) | 827 |
| **Smoking** | Non-smoker | Reference | — | Reference | — |
|  | **Smoker** | **2.17 (1.15–3.98)** | **14** | **2.24 (1.09–4.54)** | **26** |
| **Hypertension** | Absent | Reference | — | Reference | — |
|  | Stage 1 | 1.24 (0.37–4.23) | 724 | 1.15 (0.33–4.07) | 829 |
|  | Stage 2 | 1.35 (0.45–4.32) | 596 | 1.39 (0.45–4.61) | 572 |
|  | Stage 3 | 0.73 (0.31–2.02) | 0.500 | 0.78 (0.30–2.30) | 631 |
| **Diabetes** | Absent | Reference | — | Reference | — |
|  | Present | 0.80 (0.23–2.09) | 675 | 0.80 (0.23–2.20) | 693 |
| **Ischemic heart** | Absent | Reference | — | Reference | — |
|  | Present | 0.93 (0.43–1.84) | 838 | 0.99 (0.44–2.06) | 979 |
| **mRS at Disch.** | mRS 3-6 | Reference | — | Reference | — |
|  | mRS 0-2 | 0.68 (0.35–1.45) | 293 | 0.73 (0.36–1.59) | 395 |
